# Screening for Future Cardiovascular Disease Using Age Alone Compared with Multiple Risk Factors and Age

**DOI:** 10.1371/journal.pone.0018742

**Published:** 2011-05-04

**Authors:** Nicholas J. Wald, Mark Simmonds, Joan K. Morris

**Affiliations:** Wolfson Institute of Preventive Medicine, Barts and the London School of Medicine and Dentistry, Queen Mary University of London, London, United Kingdom; University of Modena and Reggio Emilia, Italy

## Abstract

**Background:**

Risk factors such as blood pressure and serum cholesterol are used, with age, in screening for future cardiovascular disease (CVD) events. The value of using these risk factors with age compared with using age alone is not known. We compared screening for future CVD events using age alone with screening using age and multiple risk factors based on regular Framingham risk assessments.

**Methods:**

Ten-year CVD risk was estimated using Framingham risk equations in a hypothetical sample population of 500,000 people aged 0–89 years. Risk estimates were used to identify individuals who did and did not have a CVD event over a ten-year period. For screening using age alone (age screening) and screening using multiple risk factors and age (Framingham screening) we estimated the (i) detection rate (sensitivity); (ii) false–positive rate; (iii) proportion of CVD-free years of life lost in affected individuals with positive results (person-years detection rate); and (iv) cost per CVD-free life year gained from preventive treatment.

**Results:**

Age screening using a cut-off of 55 years detected 86% of all first CVD events arising in the population every year and 72% of CVD-free years of life lost for a 24% false-positive rate; for five yearly Framingham screening the false-positive rate was 21% for the same 86% detection rate. The estimated cost per CVD-free year of life gained was £2,000 for age screening and £2,200 for Framingham screening if a Framingham screen costs £150 and the annual cost of preventive treatment is £200.

**Conclusion:**

Age screening for future CVD events is simpler than Framingham screening with a similar screening performance and cost-effectiveness. It avoids blood tests and medical examinations. The advantages of age screening in the prevention of heart attack and stroke warrant considering its use in preference to multiple risk factor screening.

## Introduction

Cardiovascular disease (CVD: coronary death, non-fatal myocardial infarction, and stroke) is the commonest cause of death and a major cause of morbidity worldwide [1]. Preventive treatments should therefore be more widely used, given their efficacy, low cost, and safety [2] [3].

Guidelines recommend that primary preventive treatment be based on assessment of absolute risk of cardiovascular events using multiple risk factor algorithms such as the Framingham risk equations, which include age, sex, smoking status, diabetic status, serum cholesterol, and blood pressure [4]. Age is by far the strongest determinant of CVD risk in multiple risk factor algorithms. Offering preventive treatment to everyone over a specified age without measuring other risk factors would be a simpler screening strategy than offering preventive treatment to everyone exceeding a specified CVD risk cut-off based on multiple risk factor measurement. It would avoid the multiple risk factor measurement costs. The loss in screening performance may be small enough to warrant consideration of using age alone as the screening method of choice.

To investigate this we compared the efficacy of offering preventive treatment based on age alone (age screening) with Framingham risk estimation (Framingham screening). Using illustrative costs, we also compared the cost effectiveness of these methods.

## Methods

Screening performance was assessed by estimating the detection rate (proportion of affected individuals (those who have a first CVD event within a specified time period) with positive screening results) for a given false-positive rate (the proportion of unaffected individuals (those who do not have a first CVD event within the same specified time period) with positive results). For example, in age screening, a 60% detection rate for a 20% false-positive rate at a given age cut-off means that 60% of all individuals in a population with a first CVD event occurring over a specified time period, and 20% of individuals without a CVD event over the same time period, are at or above the age cut-off. Regardless of the specified time period, this is equivalent to detecting 60% of all individuals in a population who have a first CVD event in every calendar year.

Estimates of detection rates and false-positive rates were used to compare the performance of age screening with multiple risk factor screening. Obtaining reliable estimates for such comparisons requires a very large population with known CVD risk factor values, and the identification over 10 years of individuals who do or do not have a first CVD event (ie. distinguishing affected from unaffected). A simulation study is the appropriate method of analysis because it generates a large complete dataset that reflects the distribution of age and risk factors in a whole population.

### 1. Generating a population with known values of cardiovascular risk factors

A sample of 500,000 individuals aged from 0 to 89 was generated, having the same age and sex distributions as England and Wales (2007) [5] using Monte Carlo simulation. This sample size was sufficient to give precise estimates of screening performance (to within one decimal place). The means and standard deviations of risk factors in 10-year age and sex groups, taken from the Health Survey for England [6] (summarized in tables S1 and S2 in appendix S1), were used to classify each of the 500,000 individuals as smokers or non-smokers and diabetic or non-diabetic, and to assign values for systolic blood pressure and total and HDL cholesterol, taking their distributions to be Gaussian. In this way the distributions of cardiovascular risk factors perfectly reflected the age- and sex-specific distributions of the England and Wales population. Correlations between these risk factors, given age and sex, are low (see table S3 in appendix S1) and were taken to be zero. Left ventricular hypertrophy was excluded from the Framingham risk calculation because the Health Survey for England does not provide data on its prevalence and it is not usually included in risk factor assessments.

### 2. Determining affected and unaffected individuals

Framingham estimates of the annual risk of a first CVD event (fatal or non fatal) were calculated for each of the 500,000 individuals in the simulated population [7] for each of the next ten years of their lives, based on age, sex, and risk factor values. The risk of a first CVD event was taken to be the sum of the risks of CHD death, non-fatal myocardial infarction and stroke, calculated from the results of the report of the Framingham Heart study, in which these three outcomes were individually specified [7]. The Framingham risk equations were based on people aged 30–74; it was assumed in our analysis that the same regression coefficients applied to people aged under 30 and 75 and over, and the validity of these assumptions was tested (see 5 below). For each year of the simulated 10-year follow-up period, those individuals who would have a first CVD event (affected) in the absence of preventive treatment were identified using Monte Carlo simulation; the probability of having a CVD event in a given year being the Framingham risk estimate. Conceptually this is equivalent to spinning a roulette wheel for each individual for each year such that the proportion of reds on the wheel is exactly the same as his or her Framingham risk estimate. If the wheel turns up red, the individual is classified as having had a CVD event in that year, otherwise the individual is classified as unaffected. Individuals who died of non-CVD causes were identified using England and Wales age-specific non-CVD death rates in the same way. The expected years of CVD-free life lost were calculated by estimating the average time to the first CVD event or to death from any cause in the simulated population according to sex and age. For example, a man aged 60 has an expectation of life of 14 years without a CVD event, so if he had a CVD event at age 60, he was deemed to have lost 14 years of CVD-free life.

### 3. Estimating screening performance

In our evaluation of Framingham screening, risk assessments are performed either annually or five-yearly from age 40 until an individual's 10-year CVD risk reaches a specified level (eg. a 20%, or 1 in 5 risk over 10 years), after which time the individual remains screen-positive, with no further screening assessments. In age screening, an individual becomes screen-positive when they reach a specified age.

Applying these two approaches to the sample population we determined, for specified 10-year CVD risk cut-off levels and age cut-offs, the: (i) detection rate; (ii) false-positive rate; (iii) person-years detection rate, which is the proportion of all CVD-free years of life lost in affected individuals which is lost by those who are screen-positive, ie. CVD-free years of life lost in affected individuals with a positive result divided by the CVD-free years of life lost in all affected individuals. The person-years detection rate is lower than the detection rate for the same cut-off because CVD events in younger people lead to more years of life lost without a CVD event than events in older people. For example, three individuals aged 50, 60 and 70 have life expectancies without a CVD event of 21, 16 and 13 years respectively. If they have a CVD event at 50, 60 and 70, in age screening with a cut-off of age 55, two of these three events (at age 60 and 70) would be detected: a 67% (2/3) detection rate, but the person-years detection rate would be 58% because 29 (16+13) of the 50 years (21+16+13) of CVD-free life lost would be detected.

### 4. Comparing the costs of the screening methods

The years of CVD-free life gained were calculated on the basis that a standard dose statin and three half-standard dose blood pressure-lowering drugs administered together, regardless of an individual's cholesterol concentration or blood pressure, would prevent 80% of coronary heart disease deaths and non-fatal myocardial infarctions, and 70% of strokes [2] [8] [9] [10]. The proportional reduction in cardiovascular risk was based on the observation that this is independent of the levels of the risk factors [11] [12] [13] [14]. The estimates are based on the age group 55–69, with some attenuation of effect with increasing age that will affect all screening strategies similarly. The full impact of preventive treatment would be achieved about 2–3 years after the start of therapy because the benefits of serum cholesterol reduction take some time to be realized [8].

Cost-effectiveness was estimated by calculating the costs per CVD-free year of life gained using three illustrative costs for annual treatment (£100, £200, £400 including prescribing costs) and three illustrative costs of the screening assessments (£100, £150 or £200 per assessment). The cost of inviting people to be seen is not included because it is small and applies equally to the different methods. The cost per CVD-free year of life gained was calculated by multiplying the annual cost of treatment by the person-years of treatment plus, for Framingham screening, the cost of a Framingham risk assessment multiplied by the number of assessments, all divided by CVD free life-years gained.

### 5. Validation of methods

We tested the validity of the assumptions that the Framingham risk equations ranked risk correctly in people of all ages, including those under 35 and those 75 and over by comparing the expected performance of age screening based on the expected age-specific incidence of CVD events using the Framingham risk equation with those observed from CVD registry data in England and Wales [15] (see Appendix S1). If the screening performance of the two methods yields identical or near identical results, the methodology is validated.

## Results

**Figure 1:**
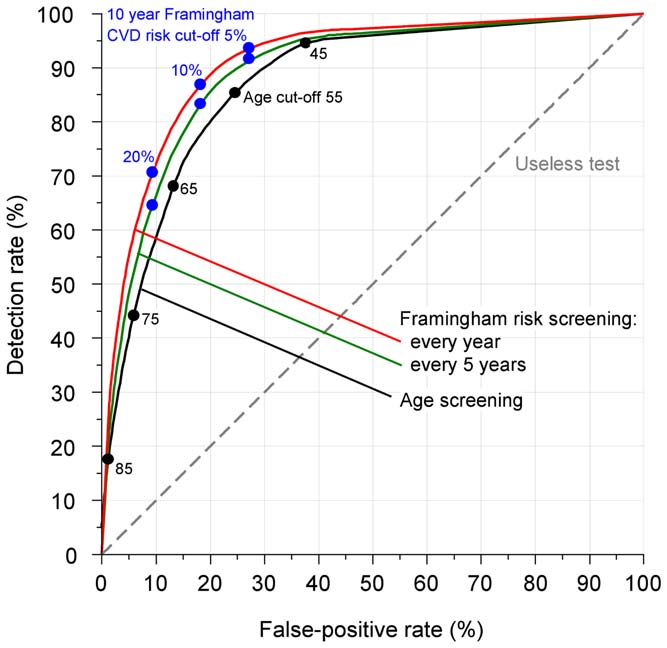
Detection rate against false-positive rate for Framingham screening and age screening showing selected age and Framingham risk cut-offs.

Figure 1 shows the detection rate plotted against the false positive rate (the receiver-operator characteristic (ROC) curve) for age screening and annual and five-yearly Framingham screening with selected age and Framingham risk cut-offs. The curves show that for a given false-positive rate the detection rate for age screening is less than for Framingham screening, but the proximity of the three curves indicates that the methods have similar screening performances. At a detection rate of 65%, for example, (achievable using five-yearly Framingham screening with a 20% 10-year CVD risk cut-off) the false positive rates are 7% for annual Framingham screening, 9% for five-yearly Framingham screening and 12% for age screening. A summary measure of screening performance is the area under the curve, 0.89 for age screening, 0.90 for five-yearly Framingham screening and 0.91 for annual Framingham screening. There are disadvantages with this measure; it conceals the different degrees of discrimination at different points along the curve, (ie. at different false-positive rates), and a useless test has a value of 0.5, not zero. Precise estimates of the screening performance shown in figure 1 are given in Appendix table S4 in Appendix S1.

**Figure 2:**
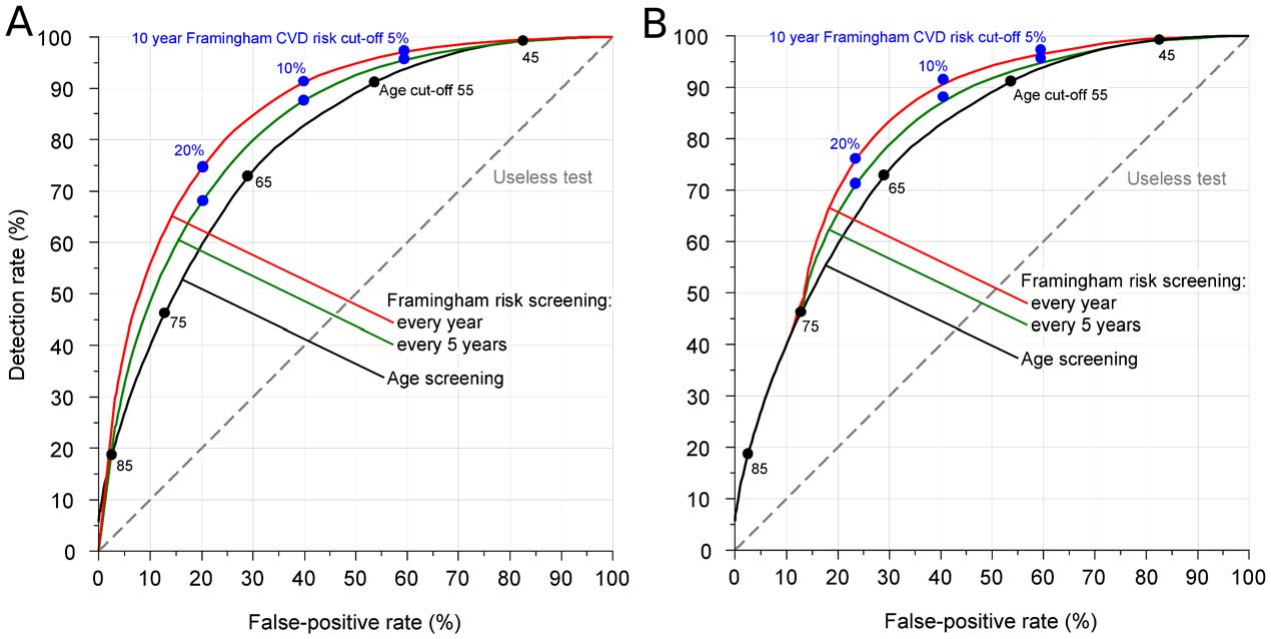
Detection rate against false-positive rate for Framingham screening and age screening: (a) in people aged 40–89 (b) as proposed by NICE (Framingham to age 74, then age screening to 89).

Figure 2a shows, in the same way as figure 1, the screening performance among people aged 40–89 (the age range that would be offered screening according to the NICE guidelines [16]). The pattern of results is similar but for the same detection rates the false-positive rates are about double because about half the unaffected population (those under 40) are excluded, almost all of whom are screen-negative. The detection rate is unchanged because there are few CVD events in people under 40. (The areas under the curves are 0.79, 0.83, and 0.86 respectively.) Figure 2b shows the screening performance of Framingham screening aged 40–74 and age screening thereafter (as proposed by NICE in the UK [16]) compared with age screening in people aged 40–89. The results are similar to those in figure 2a.

**Figure 3:**
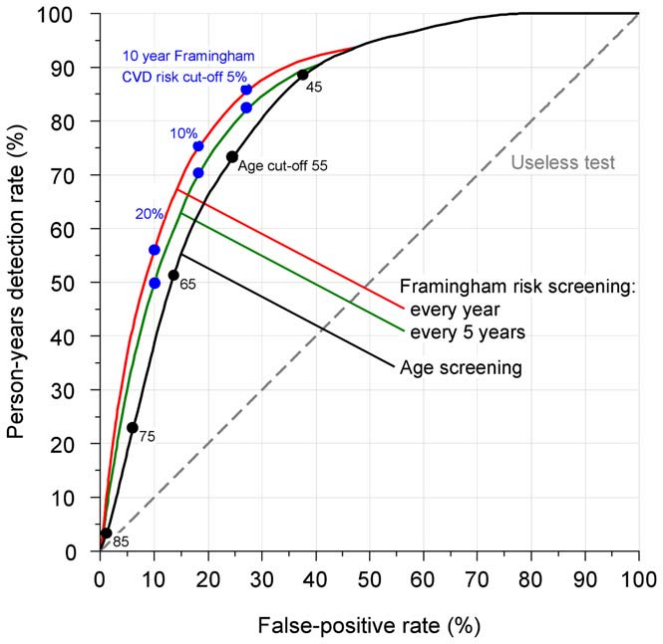
Person-years detection rate against false-positive rate for Framingham screening and age screening.

Figure 3 shows the person-years detection rate plotted against the false positive rate. The person-years detection rate is lower than the detection rate for the same false positive rate, but the pattern of results remains the same. For example, the person-years detection rate using five-yearly Framingham screening with a 20% 10-year CVD risk cut-off is 48%, so this screening strategy would identify less than half of the years of life that could be gained using preventive treatment. Precise estimates of screening performance are given in Table S5 in Appendix S1.

**Figure 4:**
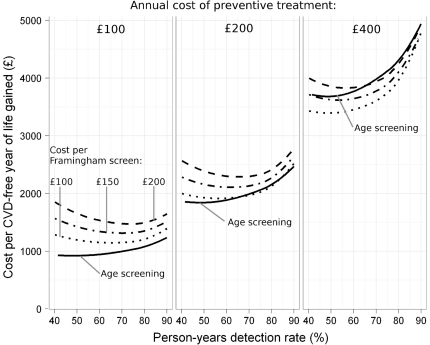
Cost per CVD-free year of life gained according to person-years detection rate and specified costs, using illustrative costs of treatment and screening given in text.

Figure 4 shows the cost per CVD-free life year gained for age screening and Framingham screening every 5 years according to person-years detection rate using the specified illustrative unit costs of treatment and screening. The cost-effectiveness of age screening and 5-year Framingham screening is similar in all the examples for a given annual treatment cost (for example, about £2,000 per CVD-free life year gained for age screening and £2,200 for Framingham screening if a Framingham screen costs £150 and the annual cost of preventive treatment is £200). The cost-effectiveness estimates, as expected, are more favourable for age screening when the treatment costs are lower and the screening costs are higher and more favourable for Framingham screening when the opposite applies, but the differences are small – typically £100 or £200 per CVD-free life year gained. Even if the cost per risk assessment were much lower, for example £50, the difference is small.

Framingham screening with annual assessments is, as expected, less cost-effective than five-yearly screening as it involves more screening assessments. For example, with an annual treatment cost of £200 and a Framingham screening cost of £150, the cost per CVD-free life year gained at a 60% person-years detection rate is £3,200 for annual screening compared with £2,100 for screening every five years. The number of screening assessments per CVD-free year of life gained would be, respectively, 23 and 6.

Table 1 summarizes the main results for Framingham screening with assessments every 5 years using the widely adopted 20% 10 year CVD risk cut-off, and the corresponding age screening results applied to achieve the same detection rate. It also shows the results of offering treatment to everyone aged 55 and over, and to everyone 50 and over, together with the corresponding Framingham screening results. Age screening with an age cut-off of 55 is equivalent to 5-yearly Framingham screening using a 10 year risk cut-off of 8%. Both methods yield person-years detection rates of 72% (86% detection rate), with age screening having a 24% false-positive rate compared with 21% with Framingham screening and a cost of £2000 per CVD-free year of life gained, instead of £2200.

**Table 1:** Main results of (i) Framingham screening every 5 years using a 20% 10-year CVD risk cut-off and (ii) age screening with a cut-off of 55 years.

Screening method	Detection rate (%)	Person-years detection rate (%)*	Screening cut-off	False-positive rate (%)	Person-years of treatment per CVD-free life year gained	Cost per CVD-free year of life gained**
**Framingham screening every 5 years using a 20% 10-year CVD risk cut-off**	66	45	1 in 5 (20%) 10-year risk	9	7	£2,200
**Age screening every year to achieve the same detection rate**	66	45	66 years	12	9	£1,800
**Age screening using a cut-off of 55 years**	86	72	55 years	24	10	£2,000
**Framingham screening every 5 years to achieve the same detection rate**	86	72	1 in 12 (8%) 10-year risk	21	9	£2,200
**Age screening using a cut-off of 50 years**	91	81	50 years	31	11	£2,200
**Framingham screening every 5 years to achieve the same detection rate**	91	81	1 in 20 (5%) 10-year risk	27	9	£2,300

*Risk and age cut-offs differ marginally between detection rate and person-years detection rate, but the difference is less than 1% or 1 year.

**Based on £200 annual cost of preventive treatment and £150 cost of a Framingham risk assessment.

About 90% of individuals have concordant results from Framingham screening and age screening. For example, using the above cut-offs (age 55 and 8% 10-year risk using 5-yearly Framingham screening) among affected individuals, 3% would be missed using Framingham but detected using age, and 6% would be missed using age but detected using 5-yearly Framingham. Among unaffected individuals 6% would be classified positive using age but negative using Framingham, and 4% would be positive using Framingham but negative using age. Framingham screening does not identify people at younger ages that have CVD events. Five-yearly Framingham screening with a 20% 10 year risk cut-off would miss only 5% of people over 70 who have a CVD event, but would miss 52% of people with such events in their 50's.

The age-specific risk of cardiovascular disease is higher in men than in women, so we explored the use of sex-specific age cut-offs. At a fixed detection rate the age cut-offs would be about 1–2 years younger in men and about 3–4 years older in women than the age cut-off for both sexes combined.

Sensitivity analyses were performed in respect of the cost-effectiveness estimates in Figure 3 and Table 1. We here present a summary of the results. The absolute cost estimates depend on the size of the effect of preventive treatment but the relative differences are similar. If, for example, the effect of treatment is halved the costs are doubled but the percentage difference is similar. With Framingham screening every five years at a 20% CVD risk cut-off, the cost per CVD life year gained is £3700 instead of £2200 and for age screening at the same detection rate the cost is £3300 instead of £1800. Discounting costs and benefits has little effect on the estimates because both are spread similarly over time. Adherence to screening or treatment also has little effect on the estimates because for those who do not adhere there is no cost or benefit and for those who do adhere the same costs and benefits apply. No adjustment was made for quality of life because every life-year gained without a first CVD event was taken to be of equal value.

The simulated population took account of advancing age, but ignored changes in risk factors and habits that affect risk. Allowing other risk factors to vary over the ten year follow-up period had a negligible effect on our results. The simulated population was based on Gaussian distributions of the risk factors but results are robust to changes in these distributions, for example, the results were not altered if all the risk factors were regarded as log-Gaussian. Framingham screening was taken to have started from age 40 as widely practised. Varying the age of starting Framingham screening has only a small effect on screening performance. For example starting at age 30 the screening performance is almost identical but the costs are greater. Starting at age 50 the detection rate and the false-positive rate are both about 2 percentage points less and the costs are less, but not reduced sufficiently to make such screening materially cheaper than an equivalent age screening policy.

**Figure 5:**
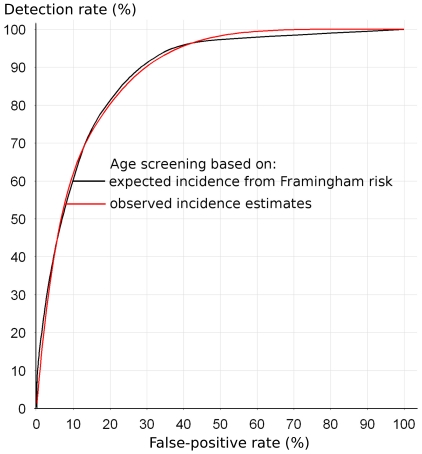
Screening performance of age screening estimated from the Framingham risk equations and from observed incidence estimates for England and Wales.

Figure 5 shows the detection rate plotted against the false-positive rate for age screening based on the expected CVD incidence from the Framingham risk assessment and that based on the observed incidence of CVD in England and Wales [15]. The two curves are essentially identical, validating our methodology. They also show that estimates of screening performance are robust to the recognized relative overestimation of risk using Framingham equations in people under about 65 [17] [18]. This arises because the ranking of people according to their risk of the same disorders is little influenced by the overall over- or under-estimation of the magnitude of their risk.

## Discussion

Our results show that age screening loses little in screening performance compared with multiple risk factor measurement methods and, with appropriately priced preventive treatment, is less expensive. Offering preventive treatment to everyone above a specified age has the advantage of simplicity. It avoids needless worry that would be caused through selecting individuals on account of the results of a personal medical assessment.

Age screening avoids the costs and time spent in connection with the measurement and explanation of risk factor levels and avoids having to issue regular invitations for blood tests and medical examinations. With age screening people are not singled out as being at risk other than on account of their age, so those taking preventive treatment are less likely to feel “abnormal” or that they have become patients and possibly given a medical diagnosis. Age screening moves the emphasis from the *assessment* of risk to the *reduction* of risk.

In multiple risk factor screening, a 10-year CVD risk cut-off of 1 in 5 (20%) has been adopted by the UK government, and recommended by the National Institute for Health and Clinical Excellence [16]. The cut-off is high for such devastating medical events as heart attacks and strokes, and does not offer preventive treatment to many people who would benefit. Over half the preventable CVD-free years of life lost would occur in people with a lower risk (see Figure 3). If this same risk were expressed as an individual having a 1 in 50 chance of a heart attack or stroke within the next 12 months, the seriousness of the situation would be more apparent and the individuals concerned would be better motivated to take steps to reduce the risk.

Preventive treatment has adverse effects but these are largely minor and reversible. With age screening a higher proportion of people are treated for the same number of cardiovascular events prevented but, given the extremely low incidence of serious adverse effects, the difference is not large enough to influence which screening method to adopt.

All methods of screening for CVD involve some people receiving preventive treatment without benefit because they die of another cause without having a CVD event, while others who would benefit do not receive treatment. For a given detection rate the proportions in these two categories are similar with Framingham screening and with screening using age alone. The prediction of CVD events using a Framingham risk assessment is relatively poor even though the Framingham equations were used to determine CVD events. This is because a Framingham risk only determines the probability of having a CVD event, not who actually has an event (eg. who, among 100 individuals with a 20% risk, are the 20 who have a CVD event).

The curves in figure 4 show that it is similarly cost-effective to deliver a screening policy designed to achieve a person-years detection rate of about 75% (a detection rate of about 85%) as it is to deliver one designed to achieve a 45% person-years detection rate, in that the number of CVD-free years of life gained for a given expenditure is similar. There is little justification for screening using a Framingham-based 20% CVD risk cut-off that has a person-years detection rate of 45% and, provided the cost of treatment is not high, is less cost-effective than the alternative of age screening using a 50 or 55 year age cut-off (see Table 1) which would achieve a detection rate of about 85%. Our results indicate that there is no practical justification for using different age cut-offs for men and women. The age cut-off, however, could be lower in people with diabetes; they have a high CVD risk and will already be aware of this.

The monetary costs we have used are illustrative and designed to provide an indication of the financial implications arising from the three methods of screening in relation to their efficacy. The costs will vary according to healthcare setting. Some costs associated with both methods have not been considered here, including the initial treatment consultation with a health professional, which does not affect the comparative costs. With Framingham screening, physicians may vary treatment according to the assessment results, and such treatment tailoring is likely to increase costs relative to age screening. Our analysis provides a reasonable indication of the relative cost-effectiveness of the two screening methods using illustrative unit costs.

We used the Framingham risk algorithm published in 1991 because it provides risk equations for the three cardiovascular outcomes we specified in this analysis (myocardial infarction, fatal coronary heart disease and stroke). Framingham risk equations published in 2008 [19] combine various cardiovascular outcomes, including, for example, angina and intermittent claudication [20]. These added outcomes are less well predicted both by age screening and by multiple risk factor measurement and consequently the detection rate is about 10 percentage points less for a 20% false-positive rate (see figure S1 in appendix S1), but our conclusions regarding the similar screening performances of age, 5-yearly, and Framingham screening still apply. They are also likely to apply to other similar algorithms, for example the Reynolds risk score [20] or QRISK2 [21] because screening performance with respect to the same clinical outcomes depends on the ranking of risk rather than the magnitude of risk. While the algorithms differ in estimating the magnitude of risk, there is little difference in the ranking of risk between individuals, mainly because in all the algorithms risk is dominated by age.

Screening *performance* was based on a population aged 0–89, but screening *programmes* would invite people aged about 40 or over for a risk assessment or simply for preventive treatment if about 50–55 or over. Using the whole population in estimating screening performance has several advantages. First, it standardizes the estimates of screening performance and avoids variation arising from the age range selected. Starting at age 40, as in Figure 2 gives similar detection rates at the same age or risk cut-off as Figure 1 but higher false-positive rates. Second, it means that all CVD events are included in the analysis to derive the estimates of screening performance, particularly CVD events in older people, in whom the disease is common and who stand to benefit considerably from preventive treatment.

A perceived limitation of this study is that it is based on statistical modelling and not on observed measurements from a cohort of individuals. The modelling is, however, based on observed data used to define the distributions of the risk factors in the population at large. The method is therefore no different from the modelling used in estimating the screening performance of, for example, Down's syndrome in pregnancy [22]. Such data-derived modelling is the preferred method of estimating and comparing screening performance because it can be based on a large enough sample to provide the necessary precision, the sample genuinely represents the population at large, and there are no missing values, with complete ascertainment of clinical events. Nonetheless it would be desirable for the estimates to be independently validated against data from a cohort study.

Causal CVD risk factors, even in combination, are poor CVD screening tests [23] [24]. To achieve even a 50% detection rate for a 5% false-positive rate, a risk factor must have a relative risk across the top and bottom quintile groups of about 100 [25]. Combining the measurement of risk factors that individually have a poor screening performance has only a small effect in improving screening performance [26]. Inappropriate emphasis on causal risk factors in CVD screening may have arisen from analyses of studies to identify causes of the disease, where the effect of age is deliberately minimized (eg. by age stratification) so that the effect of a causal risk factor is revealed. However, as we have shown, age may be, and in cardiovascuolar screening is, the dominant factor in determining risk so in assessing the value of a risk factor in screening the effect of age must be retained, and the impact of adding the risk factor to age in improving screening performance quantified.

European guidelines on the prevention of cardiovascular disease [27] recommend that “global” risk of CVD should be used to determine who should receive preventive treatment. Age alone does this. Any age can be converted into a risk; for example in Britain, at age 50 the 10-year CVD risk is 2.8%, at age 55 it is 4.5% and at age 60 it is 7.1% [15]; the risk doubles every 7.6 years, so a 90 year old person has a risk 240 times greater than a 30 year old.

In summary, CVD is common and serious. To have a major impact on its incidence a proactive cost-effective public health policy is needed. This should be designed to prevent most CVD events and should simplify access to preventive treatment without making people become patients. Age screening meets these objectives and warrants serious consideration, given its advantages over current methods of cardiovascular disease screening and prevention.

## Supporting Information

Appendix S1 (DOC)
