# Supplementary material for: Screening for Future Cardiovascular Disease Using Age Alone Compared with Multiple Risk Factors and Age
Source: PLoS One. 2011 May 4;6(5):e18742. doi: 10.1371/journal.pone.0018742 (PMC3087724; doi:10.1371/journal.pone.0018742)

**Appendix S1**

**Statistical details of the simulation**

The simulation study was conducted as follows:-

*Generation of the simulated population*

1. Random multinomial sampling was used to place each of the 500,000 individuals to one of the 18 age and sex categories, with the probability of being in each category as given in Table S2 Appendix.
2. Simulation of an individual’s assigned age was performed by taking a random sample from a uniform distribution corresponding to the age category into which the individual was placed in 1 above.
3. Simulation of smoking and diabetic status was achieved by performing a random Bernoulli trial for each individual with probability of success equal to the probability of smoking or diabetes in the appropriate age and sex category (Table S1 Appendix) excluding the 0-15 age group because of the absence of CVD events.
4. Simulation of blood pressure and cholesterol levels was performed by taking a random sample from a Gaussian distribution with the appropriate mean and standard deviation for each individual’s age and sex (Table S1 Appendix) excluding the 0-15 age group because of the absence of CVD events.

*Determination of Framingham risk*

1. The formulae and data in Anderson et al 1991[7] were used to calculate the estimated one-year and ten-year Framingham risk of CHD death, myocardial infarction and stroke using the simulated risk factors for each individual. Each individual’s age was then incremented by one year and the calculation repeated to obtain Framingham risks for the second year. The process was repeated for each year of the ten-year follow-up period.
2. The overall Framingham risk of CVD was taken to be the sum of the risks for CHD death, myocardial infarction and stroke.

*Simulation of CVD events*

1. CVD events were simulated by performing random Bernoulli trials for each individual in each year where the probability of success was equal to the one-year Framingham risk of CHD death, myocardial infarction or stroke for that individual in that year. If the random trial was a success the individual was assumed to have had the relevant CVD event in that year.
2. Prevention of events was simulated by performing a random Bernoulli trial for each simulated CVD event with probability of success equal to the preventive effect of treatment for that type of event (i.e. 0.8 for CHD death or MI, 0.7 for stroke). A success indicated that the CVD event would have been prevented if the individual were taking preventive treatment in that year.

*Screening performance*

1. Given the simulated age, sex, risk factors and CVD events and for specified age or 10-year Framingham risk cut-off the following were calculated:
   1. Whether an individual was screened in a given year
   2. Whether they were screen positive
   3. If they were a true positive, false positive, true negative or false negative
   4. If they had a CVD event prevented, and the corresponding years of CVD-free life gained (dependant on sex and age at time of prevented event)
2. From these the screening performance (detection rate, positive rate and false positive rate) and the years of treatment per life year gained and number of screening assessments per life-year gained (and hence the cost per life-year gained) were calculated.

**Determining screening performance directly from published incidence data**

The performance of age screening was determined from data on estimates of the age-specific incidence of CVD from registers published in ten-year age groups[15] and from Office for National Statistics population data.[4]

1. The 10-year incidence rates were converted into single-year estimates by fitting a log-linear model to the published incidence estimates (which for ages 25-34, 35-44, 45-54, 55-64, 65-74, and 75-84 were 2, 7, 25, 56, 105, and 270 events per year per 10,000 persons respectively).[15]

2. To determine these rates over several years “at risk”, say 10 years, we determined the risk of having a first CVD event within Y years at age A, P_Y,A_, from

P_Y,A_ = (1 – P_Y–1,A_)R_A+Y–1_ + P_Y–1,A_ with P_1,A_ = R_A_

where R_A_ is the one-year risk of an event at age A (determined from the incidence data).

3. Given these 10-year risks of CVD events by age, the numbers of events over 10 years by age were determined using the data on population by age (Table S2 appendix).

4. The performance of age screening over 10 years for specified age cut-offs was then determined by counting the number of individuals with and without events at or above specific ages, as described in the paper.

Table S1 (Appendix): Distribution of cardiovascular risk factors in the simulated population, taken from the Health Survey for England 2003^6^

| **Sex** | **Age group** | **Systolic blood pressure (mmHg)** | | **Total cholesterol (mmol/l)** | | **HDL cholesterol (mmol/l)** | | **Smokers (%)** | **Diabetic (%)** |
| --- | --- | --- | --- | --- | --- | --- | --- | --- | --- |
|  |  | **Mean** | **SD** | **Mean** | **SD** | **Mean** | **SD** |  |  |
|  |  |  |  |  |  |  |  |  |  |
| Male | 16-24 | 123.2 | 11.5 | 4.5 | 0.9 | 1.3 | 0.3 | 33 | 0.4 |
|  | 25-34 | 126.3 | 9.9 | 5.3 | 1.1 | 1.4 | 0.3 | 38 | 0.3 |
|  | 35-44 | 127.5 | 11.9 | 5.8 | 1.1 | 1.4 | 0.3 | 32 | 2.8 |
|  | 45-54 | 132.2 | 14.4 | 5.9 | 1.1 | 1.4 | 0.3 | 23 | 3.6 |
|  | 55-64 | 136.5 | 16.6 | 5.8 | 1.1 | 1.4 | 0.3 | 22 | 8.1 |
|  | 65-74 | 139.5 | 17.8 | 5.5 | 1.2 | 1.4 | 0.3 | 13 | 11.9 |
|  | 75-84 | 143.8 | 23.0 | 5.3 | 1.1 | 1.4 | 0.3 | 8 | 10 |
|  | | | | | | | | | |
| Female | 16-24 | 113.4 | 9.0 | 4.6 | 0.8 | 1.6 | 0.4 | 31 | 0.9 |
|  | 25-34 | 114.1 | 9.9 | 5.0 | 0.9 | 1.6 | 0.4 | 29 | 0.9 |
|  | 35-44 | 117.1 | 12.4 | 5.4 | 0.9 | 1.6 | 0.4 | 29 | 1.5 |
|  | 45-54 | 124.8 | 16.6 | 5.8 | 1.1 | 1.7 | 0.4 | 26 | 2.6 |
|  | 55-64 | 132.8 | 18.0 | 6.3 | 1.1 | 1.7 | 0.4 | 22 | 4.7 |
|  | 65-74 | 141.6 | 21.7 | 6.2 | 1.2 | 1.7 | 0.4 | 15 | 8.4 |
|  | 75-84 | 147.6 | 21.2 | 6.1 | 1.2 | 1.6 | 0.4 | 9 | 8.9 |

Table S2 (Appendix): Population distribution according to age and sex and non-cardiovascular disease (CVD) death rates, taken from ONS Mortality data 2005^5^

| **Sex** | **Age group** | **Percentage of population in age/sex group** | **Non-CVD death rate**  **(% per year)** |
| --- | --- | --- | --- |
|  |  |  |  |
| Male | 0-15 | 9.1 | 0.05 |
|  | 16-24 | 6.9 | 0.05 |
|  | 25-34 | 6.5 | 0.08 |
|  | 35-44 | 7.6 | 0.12 |
|  | 45-54 | 6.5 | 0.24 |
|  | 55-64 | 5.9 | 0.60 |
|  | 65-74 | 4.0 | 1.50 |
|  | 75-84 | 2.4 | 3.94 |
|  | 85-90 | 0.5 | 8.07 |
|  |  |  |  |
| Female | 0-15 | 8.7 | 0.04 |
|  | 16-24 | 6.5 | 0.02 |
|  | 25-34 | 6.5 | 0.04 |
|  | 35-44 | 7.7 | 0.08 |
|  | 45-54 | 6.6 | 0.19 |
|  | 55-64 | 6.1 | 0.46 |
|  | 65-74 | 4.4 | 1.08 |
|  | 75-84 | 3.3 | 2.88 |
|  | 85-90 | 1.0 | 6.07 |

Table S3 (Appendix): Age-specific correlation coefficients for cholesterol and systolic blood pressure in men

| Age | Number of Men | Correlation coefficient (r) | | |
| --- | --- | --- | --- | --- |
|  |  | Systolic BP and Total Cholesterol | Systolic BP and HDL Cholesterol* | Total Cholesterol and HDL Cholesterol* |
| 35-44 | 9074 | 0.1276 | 0.0014 | -0.0259 |
| 45-54 | 8070 | 0.0746 | 0.0001 | 0.0139 |
| 55-64 | 4049 | 0.0984 | 0.0656 | 0.0551 |

Table S3(b) (Appendix): Associations between smoking and diabetes status and cholesterol and systolic blood pressure in men.

| Age group | Non Smokers | Smokers | Non – diabetics | Diabetics |
| --- | --- | --- | --- | --- |
|  | Mean (number of men) | | | |
|  | | | | |
| Total Cholesterol (mmol/l) | | | | |
| 35-44 | 6.5 (5244) | 6.6 (3831) | 6.5 (8957) | 6.5 (118) |
| 45-54 | 6.8 (4717) | 6.8 (3355) | 6.8 (7874) | 6.8 (198) |
| 55-64 | 6.8 (2347) | 6.8 (1702) | 6.8 (3907) | 6.5 (142) |
|  | | | | |
| HDL Cholesterol (mmol/l)* | | | | |
| 35-44 | 1.4 (2449) | 1.3 (1739) | 1.3 (4164) | 1.3 (24) |
| 45-54 | 1.4 (1929) | 1.3 (1191) | 1.3 (3069) | 1.3 (51) |
| 55-64 | 1.4 (933) | 1.4 (605) | 1.4 (1505) | 1.4 (33) |
|  | | | | |
| Systolic blood pressure (mmHg ) | | | | |
| 35-44 | 128.4 (5243) | 129.1 (3833) | 128.6 (8957) | 132.7 (119) |
| 45-54 | 132.7 (4719) | 132.2 (3354) | 132.4 (7873) | 137.9 (200) |
| 55-64 | 139.9 (2347) | 138.3 (1702) | 139.1 (3907) | 143.4 (142) |

* limited to men with an HDL measurement (4188, 3120 and 1538 respectively)

(Unpublished results from the BUPA study)

Table S4 (Appendix): False positive rates and detection rates with screening cut-offs for age screening and Framingham screening

|  | **Framingham screening** | | **Age screening** | **Framingham screening**  **(10 year risk)** | | **Age screening (years)** |
| --- | --- | --- | --- | --- | --- | --- |
|  | **every year** | **every 5 years** |  | **every year** | **every 5 years** |  |
|  |  | | |  | | |
| **False positive rate (%)** | **Detection rate (%)** | | | **Screening cut-off** | | |
|  |  |  |  |  |  |  |
| 5 | 55 | 48 | 40 | 30% | 30% | 76 |
| 10 | 72 | 66 | 59 | 19% | 19% | 69 |
| 20 | 88 | 85 | 80 | 9% | 9% | 59 |
| 30 | 95 | 93 | 91 | 4% | 4% | 50 |
| 40 | 97 | 96 | 96 | 1% | 1% | 43 |
|  |  |  |  |  |  |  |
| **Detection rate (%)** | **False positive rate (%)** | | | **Screening cut-off** | | |
|  |  |  |  |  |  |  |
| 60 | 6 | 8 | 11 | 27% | 23% | 68 |
| 70 | 9 | 11 | 14 | 20% | 17% | 64 |
| 80 | 14 | 17 | 20 | 14% | 11% | 59 |
| 90 | 21 | 25 | 29 | 8% | 6% | 51 |
| 95 | 30 | 37 | 39 | 4% | 2% | 44 |

Table S5 (Appendix): False positive rates and person-years detection rates with screening cut-offs for age screening and Framingham screening

|  | **Framingham screening** | | **Age screening** | **Framingham screening**  **(10 year risk)** | | **Age screening (years)** |
| --- | --- | --- | --- | --- | --- | --- |
|  | **every year** | **every 5 years** |  | **every year** | **every 5 years** |  |
|  |  | | |  | | |
| **False positive rate (%)** | **Person-years detection rate (%)** | | | **Screening cut-off** | | |
|  |  |  |  |  |  |  |
| 5 | 37 | 30 | 20 | 30% | 30% | 76 |
| 10 | 56 | 50 | 39 | 19% | 19% | 69 |
| 20 | 77 | 73 | 66 | 9% | 9% | 59 |
| 30 | 88 | 85 | 81 | 4% | 4% | 50 |
| 40 | 93 | 91 | 91 | 1% | 1% | 43 |
|  |  |  |  |  |  |  |
| **Person-years detection rate (%)** | **False positive rate (%)** | | | **Screening cut-off** | | |
|  |  |  |  |  |  |  |
| 40 | 6 | 7 | 10 | 28% | 24% | 69 |
| 50 | 8 | 10 | 13 | 22% | 19% | 65 |
| 60 | 11 | 14 | 16 | 17% | 14% | 62 |
| 70 | 16 | 18 | 22 | 12% | 10% | 57 |
| 80 | 23 | 9 | 29 | 7% | 6% | 51 |
| 90 | 32 | 40 | 39 | 3% | 1% | 44 |

**Figure S1 Appendix S1: Framingham 2008: Detection rate against false-positive rate using the revised 2008 Framingham risk equations (Figure 1 in main text is based on 1991 equations)**


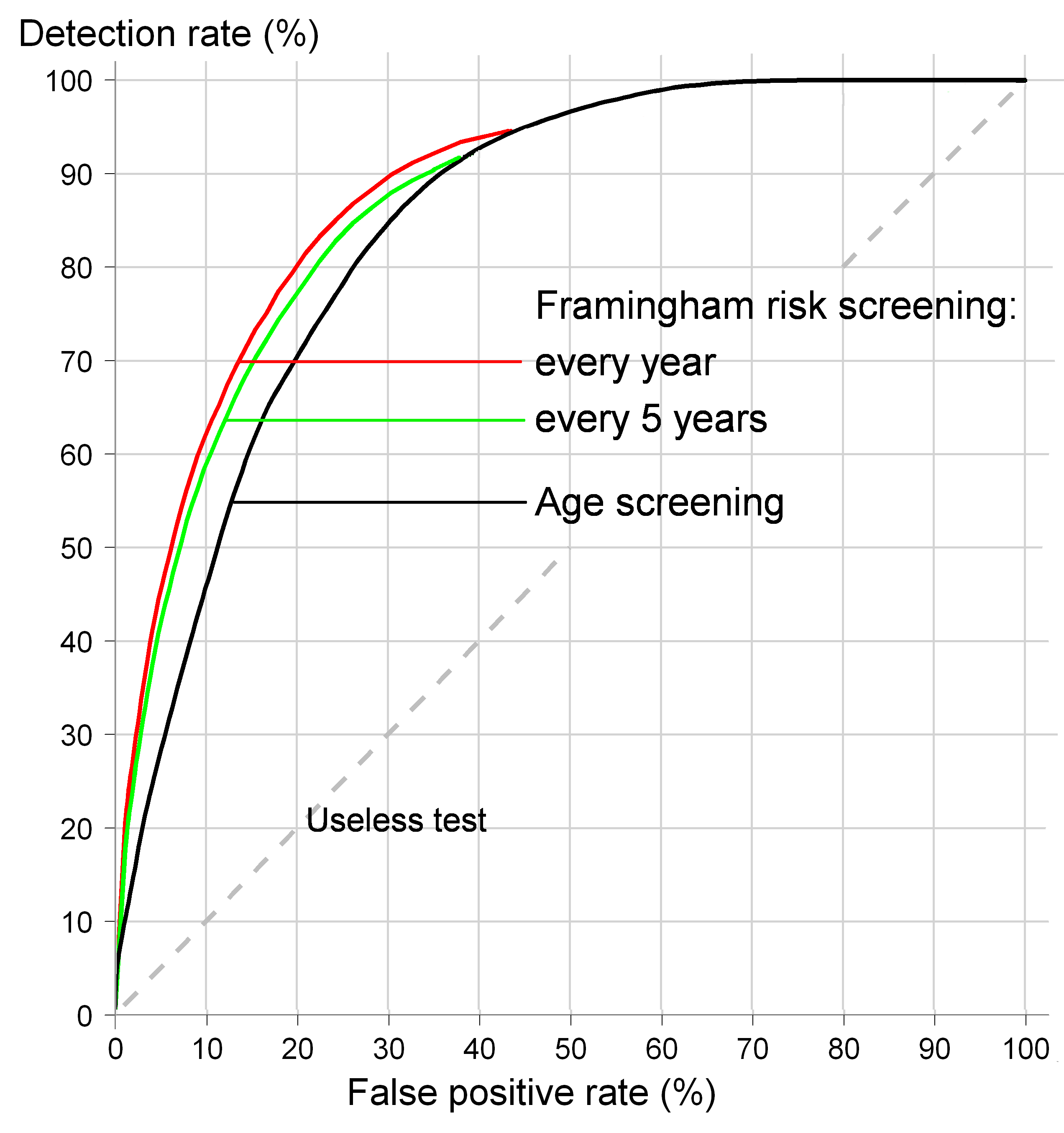

Supplement: Appendix S1 — (DOC) [file pone.0018742.s001.doc]
